# Strategies for the Accurate Measurement of the Resonance Frequency in QCM-D Systems via Low-Cost Digital Techniques

**DOI:** 10.3390/s22155728

**Published:** 2022-07-31

**Authors:** Tommaso Addabbo, Ada Fort, Elia Landi, Riccardo Moretti, Marco Mugnaini, Valerio Vignoli

**Affiliations:** Department of Information Engineering and Mathematics, University of Siena, 53100 Siena, Italy; addabbo@diism.unisi.it (T.A.); ada@diism.unisi.it (A.F.); landi@diism.unisi.it (E.L.); mugnaini@diism.unisi.it (M.M.); vignoli@diism.unisi.it (V.V.)

**Keywords:** QCM sensors, QCM-D measurement technique, digital frequency meter

## Abstract

In this paper, an FPGA (Field Programmable Gate Array)-based digital architecture for the measurement of quartz crystal microbalance (QCM) oscillating frequency of transient responses, i.e., in QCM-D (QCM and Dissipation) applications, is presented. The measurement system is conceived for operations in liquid, with short QCM transient responses due to the large mechanical load. The proposed solution allows for avoiding the complex processing systems typically required by the QCM-D techniques and grants frequency resolutions better than 1 ppm. The core of the architecture is a reciprocal digital frequency meter, combined with the preprocessing of the QCM signal through mixing operations, such as a step-down of the input frequency and reducing the measurement error. The measurement error is further reduced through averaging. Different strategies are proposed to implement the proposed measurement solution, comprising an all-digital circuit and mixed analog/digital ones. The performance of the proposed architectures is theoretically derived, compared, and analyzed by means of experimental data obtained considering 10 MHz QCMs and 200 μs long transient responses. A frequency resolution of about 240 ppb, which corresponds to a Sauerbrey mass resolution of 8 ng/cm^2^, is obtained for the all-digital solution, whereas for the mixed solution the resolution halves to 120 ppb, with a measurement time of about one second over 100 repetitions.

## 1. Introduction

Quartz Crystal Microbalances (QCMs) are sensors based on piezoelectric electromechanical resonators, which interact with the environment and change their resonant behavior due to different interaction mechanisms. QCMs-based sensing systems have a wide range of applications, such as gas sensing, humidity sensing, particle sensing, biosensors for a variety of biological targets, film growth monitoring in electrochemical deposition and liquid viscosity sensing [1,2,3,4,5,6,7,8,9,10,11,12]. The use of QCM has some consolidated application fields but is still a subject for many research works aiming at improving performance from different points of view. In particular, the most active research topics, nowadays, concern the application of QCMs in reproductive medicine and point-of-case diagnostics [13,14,15], and the use of novel sensing layer material, the development of new applications and of new models for the interpretation of measurement data and the design of improved measurement systems and techniques [16].

Focusing on this last topic, i.e., the development of measurement systems, to evaluate the resonator behavior, several electronic systems and techniques have been proposed, relying on resonance frequency measurement, which typically exploits electronic quartz oscillator circuits [17,18] or on the evaluation of other quantities such as those based on the measurement of the QCM electrical impedance [19,20,21] or on the induced phase shift [22,23,24]. Some notable examples are the QCM 200 [25], which is a commercial device that realizes the frequency measurement using continuous oscillatory circuits which exploit automatic gain control circuitry; the 0-phase electronic circuit for QCM operation in highly viscous environments implemented by Avramov [26], which recovers the quartz parameters via an $S_{21}$ transmission measurement after the tuning of the quartz via an LC network to reduce the effects of electrical loading and parasitic contributions given by the electrical loading of the measurement system; or the OPENQCM Q-1 with dissipation module [27], another commercial device that analyzes the resonance curve characteristics using a network analyzer in a wide frequency range, allowing to also detect and monitor overtones.

Focusing on the direct resonance frequency measurement, a more versatile widespread technique is the Quartz Crystal Microbalance with Dissipation Monitoring (QCM-D) [28,29,30,31,32,33,34,35,36,37]; this technique is based on the simultaneous measurement of the sensor resonance frequency and of its dissipation factor (i.e., the reciprocal of the quartz quality factor) during a transient response triggered by short electric excitations.

The challenges related to the design of electronic read-out circuits for QCM sensors are related to the involved complex sensing mechanisms. In general, the sensing principle of QCMs relies on the variation of the electromechanical resonator behavior due to the mechanical load exerted by the environment on the vibrating quartz, or, in other words, caused by the acoustic coupling between the quartz and the media in contact with its surfaces. The most common quartzes used for QCM, which are shear wave bulk resonators, usually operate at 5 MHz or 10 MHz, and the pristine devices, surrounded by air, are resonators with very high-quality factors (in the order of some tens of thousands), so that their transient response due to impulsive stimuli or to non-zero initial conditions lasts for some milliseconds. For high Q resonators, the design of the read-out circuit is not critical [38].

When used as sensors, quartzes are functionalized by the deposition of a sensing layer over one of their surfaces, and, especially for bio-sensing, they can be immersed in liquid. In these cases, the elastic wave responsible for the vibration is transmitted also to the sensing film and/or leaks in the liquid. The coupling with the film and/or the environment changes both the resonance frequency and the quality factor of the quartz. If a target is absorbed by the sensing layer or if the liquid changes its composition, the QCM resonant behavior changes as well, and this is the base of sensing with QCMs; it can be shown that the frequency changes as a function of the mass of the ad-layer and of the viscoelastic characteristics of the media surrounding the quartz, whereas the Q factor mainly depends on the viscosity of the media in contact with the resonant system. In in-liquid applications, due to the liquid viscosity, the quality factor of the resonant system drops by about an order of magnitude (or more), so the transient response duration decreases down to hundreds of microseconds. For low Q-factor QCM applications, the design of the readout and measurement electronics becomes critical; moreover, the measurement requirements in terms of measurement range and resolution are very demanding: in most applications, the expected maximum relative variation of the resonance frequency is in the order of hundreds of ppms (some kHz for 10 MHz QCM) and the required frequency relative resolution is in the order of 100 ppb or less, (1 Hz or less for a 10 MHz quartz); moreover, it can be shown that, especially for low Q situations, the measurement of the ‘resonance frequency’ through the monitoring of the oscillation frequency of the transient response has many advantages in terms of accuracy, because other techniques lead to systematic errors, which increase when the Q-factor decreases [38]. Therefore, QCM-D is a very convenient measurement technique for these cases. Nevertheless, since the transient response becomes short, the frequency assessment resolution can drop unless suitable measurement solutions are adopted [29,39,40,41,42,43].

To evaluate the transient oscillation frequency and decay time, the response signal is typically passed through an analog-to-digital converter and digitally processed [44,45]. Digital processing methods include direct model fitting (both in time and in frequency domains) [46,47,48], Discrete Hilbert Transform [49], parametric modeling [50,51], or Discrete Fourier Transform [52,53]; what all these methods have in common is the need for a complex processing system.

Even if many low-complexity and low-cost measurement systems with very good metrological characteristics have been proposed in the literature or are commercially available, which exploit oscillators and measure the frequency of a sine wave produced by a feedback amplifier with the quartz embedded in the loop [17], or zero-phase lock-in circuits [54], no relevant low-cost accurate solution has yet emerged regarding the QCM-D technique [55,56].

In this paper, we approach the problem of QCM-D measurement systems by proposing a low-cost, FPGA (Field Programmable Gate Array)-based frequency meter device for exponentially decaying sinusoidal QCM-D response signals. The system combines the well-known reciprocal frequency counter architecture with the preprocessing of the QCM-D signal through mixing operations, such as step-down the signal frequency and improving the measurement accuracy.

The paper is organized as follows. In Section 2, the background and motivation of the measurement technique are described. In Section 3 the frequency measurement technique is described, also providing some design considerations related to the measurement constraints. In Section 4, an application case study is provided. In Section 5, the optimized device architecture, implementing the response signal processing, and the frequency measurement, is presented; moreover, the experimental results are presented and analyzed. Finally, in Section 6, the conclusions are drawn.

## 2. Background and Motivation

Quartz Crystals Microbalances (QCMs) are sensors obtained sandwiching a thin piezoelectric crystal between two conducting electrodes.

In this discussion, the considered quartz is an AT-cut quartz, which vibrates in shear thickness mode, with active area A, thickness t, density $\rho_{q},\;$electric permeability ϵ, piezoelectric coefficient $e_{53}$. When a gas surrounds the pristine QCM, the boundary conditions for the acoustic field at the quartz surfaces are characterized by a null shear stress. Exploiting these conditions, the mechanical impedance of the quartz can be found, and, in turn, it can be transformed into an electrical impedance due to the piezoelectric electromechanical coupling, which can be written as follows:

(1) $$Z_{e}=\frac{1}{j\omega C_{0}}\;\left[1-\frac{K^{2}vA}{\omega t\;}2\tan\left(\frac{\omega }{v_{q}}\frac{t}{2}\right)\right]\;,$$

where ω is the angular frequency, $C_{o}=\frac{\epsilon A}{t}$ is the electrical capacitance of the quartz, whereas $v_{q}=\sqrt{\frac{\mu_{q}^{*}}{\rho_{q}}}$, is the shear wave speed in the quartz, and $\mu_{q}^{*}=\mu_{q}+j\omega \eta_{q}$ is the complex shear modulus, being $\mu_{q}$ the shear modulus and $\eta_{q}$ the viscosity of the quartz. Finally, $K=\frac{e_{53}^{2}}{\mu_{q}\;\epsilon }$ is the piezoelectric coupling coefficient. (1) represents an impedance with an infinite number of resonances.

The behavior of the impedance in (1) around the first resonance frequency can be approximated with a lumped parameter network, where the quartz is represented by the parallel of the electrical capacitance and a motional branch consisting of a series resonant circuit, i.e., by the following impedance [39,40]:

(2) $$Z_{m}=R_{m}+j\omega L_{m}+\frac{1}{j\omega C_{m}}-\frac{1}{j\omega C_{0}}\;,$$

where:

(3) $$C_{m}=\frac{9K^{2}C_{0}}{\pi^{2}\;};L_{m}=\frac{t^{2}\rho_{q}}{8C_{0}K^{2}\mu_{q}\;};R_{m}=\frac{\pi^{2}\eta_{q}}{8C_{0}K^{2}\mu_{q}}\;.$$

**Figure 1:**
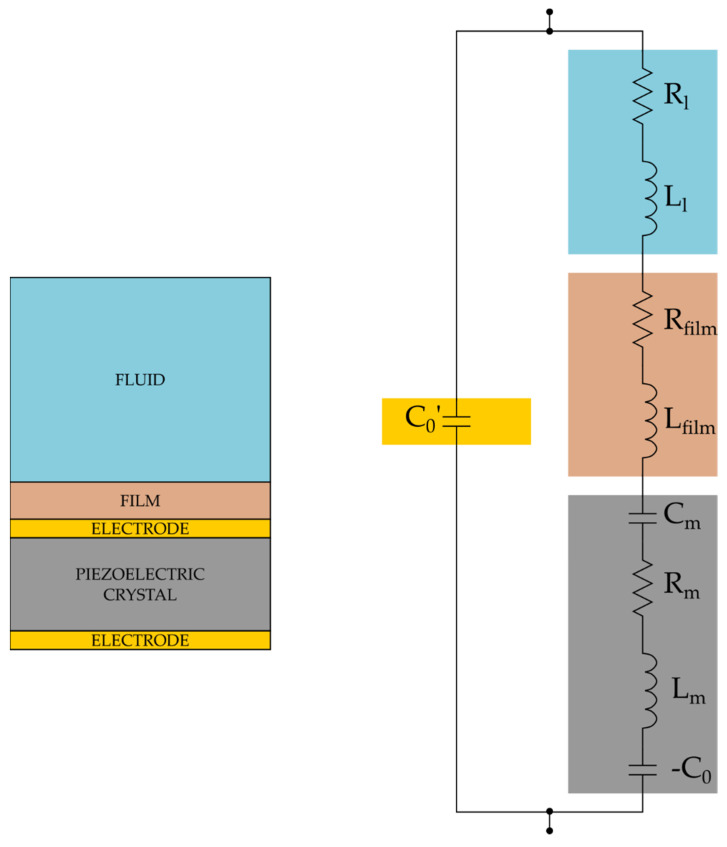
Butterworth-Van-Dyke model of a loaded QCM.

The corresponding equivalent circuit, neglecting the effect of the last term in (2), being $C_{0}\gg C_{m}\;$, is named Butterworth Van Dyke (BVD) model and is shown in Figure 1. In the BVD model, the pure electric branch formed by the capacitance $C_{0}$ is in parallel with the motional branch ($L_{m}$, $R_{m}$ and $C_{m}$), which well approximates the mechanical behavior of the quartz, and is characterized by a series resonance frequency, given by the following equation:

(4) $$f_{s}=\frac{1}{\sqrt{L_{m}C_{m}}}\;.$$

(4) shows that the series resonance frequency depends solely on the mechanical characteristics of the resonator.

On the other hand, the parallel resonance frequency $f_{p}$ is dependent on the parallel capacitor $C_{0}^{\prime }=C_{0}+C_{p}$, given by the $C_{0}$ of the quartz, as defined above, in parallel to any parasitic capacitance connected to the two electrodes, comprising the one offered by wiring or by the front-end electronics $C_{p}$:

(5) $$f_{p}=\frac{1}{2\pi \sqrt{L_{m}\frac{C_{m}C_{0}\prime }{C_{m}+C_{0}\prime }\;}}\;.$$

Finally, the Q-factor of the quartz can be written as follows:

(6) $$Q=\frac{1}{R_{m}}\sqrt{\frac{L_{m}}{C_{m}}}\;.$$

The QCM used as a sensor, either due to the presence of a functionalization layer or to the presence of a non-ideal fluid (e.g., a Newtonian fluid), has a surface in contact with a material that supports shear waves, and this situation corresponds to a change of the boundary conditions at one of the quartz surfaces. Therefore, the acoustic field inside the quartz changes, and this is reflected in a transformation of the equivalent circuit model.

In general, the equivalent electric impedance of a film deposited on the QCM film can be written as follows:

(7) $$Z_{film}=\frac{V}{I}=j\frac{t^{2}}{4Ae_{53}^{2}}\sqrt{\rho_{f}\mu_{f}^{*}}\tan\frac{\omega h}{v_{A}}\;,$$

where $v_{A}=\sqrt{\frac{\mu_{f}^{*}}{\rho_{f}}}$, is the complex shear wave speed in the film, and $\mu_{f}^{*}=\mu_{f}+j\omega \eta_{f}$ is the complex shear modulus, being $\eta_{f}$ the film viscosity and $\mu_{f}$ the shear modulus.

Considering always to work at frequencies much lower than the resonance of the film, in the lumped parameter equivalent network, the impedance of the film is usually approximated by a resistance (for the real part of $Z_{film}$) and an inductance (for the imaginary part of $Z_{film}$), placed in series to the motional branch of the BVD circuit [57].

The presence of $Z_{film}$ in the electrical circuit models the mechanical load and obviously shifts the series resonance frequency, such that the QCM sensor can be used to detect different characteristics of the added films depending on the applications; moreover, the presence of the film also affects the Q-factor of the resonant system.

In case the film viscosity is equal to 0 (purely elastic medium), the electric impedance is a pure positive reactance (until the resonance) therefore, it behaves as an inductive load; moreover, if the layer is thin the effect of the film on the series resonant frequency is the one described by the Sauerbrey equation [58], and the series frequency shift depends solely on the film (ad-layer) mass. So, in a situation such as this: in the presence of thin elastic films or rigid films QCMs behave as pure mass sensors and a negligible effect on the Q factor is seen.

On the other hand, if the film has a viscous behavior there is also a large resistive component in series appearing in the motional branch, the magnitude of which depends on the phase of the complex wave speed.

We can consider as the limit case a film composed of a Newtonian fluid, where the phase of the complex wave speed is equal to 45°, since and $v_{A}=\sqrt{\frac{j\omega \eta_{f}\;}{\rho_{f}}}$.

Note that (7) (asymptotically with the fluid layer thickness h going to ∞) describes also the impedance loading the quartz in in-liquid measurements $(Z_{l}$, with $Z_{film}\to Z_{l}$). In fact, when the thickness of the layer overcomes a certain limit the effect of the wave reflected by the upper surface of the film is negligible at the lower surface of the film in contact with the quartz and the load tends to the impedance provided by a semi-infinite space of Newtonian fluid. For fluid films and half spaces, the resistive load is very large, and the Q factor of the resonant system decreases dramatically, typically by at least one order of magnitude.

It must be noted that in some applications the sensing mechanism depends on the interaction of the QCM with individual particles (e.g., bacteria) if the size of the adsorbed objects, which form an ad-layer, is larger than the wavelength. In this case, the impedance that must be added in series to the motional branch of the BVD takes a different form from (7) and depends on the particle concentration, on the geometry and density of the particles, the density of the fluid surrounding the particles, and on the strength of the elastic bond of the particle to the surface [59,60].

The basic principle of the QCM-D technique is to excite the QCM with a burst signal, whose frequency is close to its resonance; this excitation triggers a sufficiently large transient response of the quartz which is an exponentially decaying response [20,21,22,23]. The response can be written as:

(8) $$u\left(t\right)=U_{0}e^{-t/\tau }\sin\left(2\pi f_{q}t+\phi \right)\;,$$

where $U_{0}$ is the amplitude of the initial oscillations, $f_{q}$ is the response oscillation frequency and τ is the decay time constant. The response oscillation frequency is independent of the excitation frequency; however, the nearer the excitation frequency to the response one, the higher the response amplitude $U_{0}$, therefore increasing the measurement signal-to-noise ratio [39].

Assuming to short circuit the quartz to measure the short circuit current signal and to evaluate the signal oscillation frequency $f_{q}$ and decay time constant τ, it is possible to estimate the quartz quality factor and series resonance frequency using the following equations [38]:

(9) $$Q=\pi f_{q}\tau \;,$$

(10) $$f_{s}=\frac{f_{q}}{\sqrt{1-\frac{1}{4Q^{2}}}}\;.$$

Therefore, if succeeding in measuring the transient short circuit current, the measurement of the transient signal oscillation frequency and of the time constant measurements allows the assessment of the series resonance frequency, which, as can be observed in (4), is independent of loading parasitic capacitances and directly provides information on the electromechanical properties of the resonant system and/or on the mass of the adlayer.

This explains the motivation of QCM-D measurement techniques, which in summary can separate the information related to the imaginary part of the impedance (that can give information also on the mass of the ad-layer as pointed out in the detailed discussion) and to the dissipative behavior of the ad-layer or surrounding fluid and provide estimations independent from parasitic capacitances due to the insertion of the quartz in the measurement circuit.

The critical issues are related to the need of developing front-end electronics characterized by low input impedance, behaving as a current amplifier, limiting the load effects on both the time constant and the frequency value [38].

Moreover, since the measured signal is a vanishing oscillation, which for QCM in gas sensing applications lasts tens of thousands of cycles (e.g., some milliseconds for QCM at 10 MHz) whereas in in-liquid applications few thousands of cycles (e.g., about 200 μs for QCM at 10 MHz), the frequency $f_{q}$ must be estimated in a short time window with measurement accuracies that can be relatively high (typically around tens of ppb in gas applications and ppm in in-liquid applications).

## 3. Frequency Measurement Technique

To perform the digital measurement of a signal frequency, frequency counters are commonly employed. These well-known devices are generally composed by three elements:

- a gate counter, that defines the measurement time duration;
- a pulse counter, that provides the frequency measurement as the number of pulses counted during the measurement time;
- a reference clock signal, whose frequency is used to convert the counted pulse number into a frequency value.

**Figure 2:**
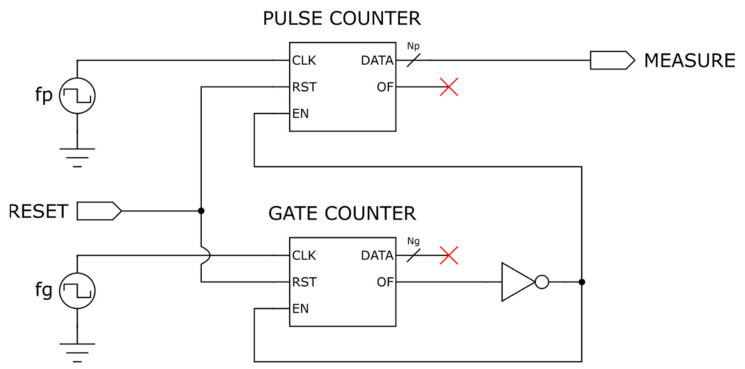
Generic structure of a frequency counter.

The generic structure of a frequency counter is shown in Figure 2. When the device is enabled (RESET = ‘0’), the gate and the pulse counters start counting, respectively, with frequency $f_{p}$ and $f_{g}$, until the gate counter reaches the overflow condition (OF = ‘1’). The overflow disables the counters, providing as output (MEASURE) the count reached by the pulse counter.

The described architecture can be configured to perform two kinds of frequency measurement.

If we connect the reference clock signal to the gate counter and the signal to be measured to the pulse counter, we perform a direct frequency measurement. In this case $f_{g}=f_{clk}$ is the reference clock frequency, while $f_{p}=f_{x}$ is the input signal frequency, being $N_{g}$ the bit length of the gate counter, and $N_{p}$ the bit length of the pulse counter. Setting the gate counter to count to $n_{g}\leq 2^{N_{g}}$ and defining with $n_{p}\leq 2^{N_{p}}\;$the number of counts reached by the pulse counter at the end of a measurement, the input signal frequency measure ${\hat{f}}_{x}$ is given by:

(11) $${\hat{f}}_{x}=\frac{n_{p}}{n_{g}}f_{clk}\;.$$

Instead, if we connect the reference clock signal to the pulse counter $f_{p}$ = $f_{clk}$ and the signal to be measured to the gate counter, $f_{g}=$ $f_{x}$, we perform a direct period (or reciprocal frequency) measurement. Using the same notation given for the direct frequency measurement, in this case, the input signal frequency measure is given by:

(12) $${\hat{f}}_{x}=\frac{n_{g}}{n_{p}}f_{clk}\;.$$

According to (11), the measurement resolution of a direct frequency counter depends on $n_{g}/f_{clk}$, i.e., the measurement time duration; this means that to achieve a high-frequency resolution, we need to perform a long measurement; this condition does not fit our application, since the QCM-D response signal (8) has a limited time duration, and therefore we must design our device implementing a reciprocal frequency counter.

The uncertainty of the proposed technique must be evaluated by considering two sources of uncertainty: one related to the measurement method, and to the process of counting therefore discretizing time, and the other related to the presence of measurement electronic noises (e.g., white noise, phase jitter) injected and caused by the implementation of the measurement hardware through real components.

The first contribution can be considered at first as a systematic error, assuming a deterministic phase difference between the gate, pulse and reset input signals. We call this contribution count error; the count error magnitude is related to the ratio between the clock period and the measurement time (intended as the time required by the gate counter to reach overflow), therefore it decreases as the measurement time increases. Afterward, considering the phase difference randomness, it can be shown that this error can be treated as a stochastic process, with maximum amplitude related to the systematic error cited above; it can be shown that this stochastic process, on the whole, is white and zero mean, therefore averaging consequent measurement results allows for reducing the uncertainty due to its effect.

The other source of uncertainty, which is the electronic noise injected by the circuit, is mitigated by the choice of quality components (e.g., low noise amplifiers, stable oscillators) and by carefully designing the front-end electronics and can be counteracted by averaging repeated measurement results at the expenses of measurement time.

Focusing on the count error, referring to the frequency counter scheme in Figure 2, the count error depends both on the gate and the pulse counters.

The gate counter is supposed to count from 0 to $n_{g}$, generating, therefore, the overflow after a time equal to $n_{g}/f_{x}$; however, the frequency counter RESET transition instant from ‘1’ to ‘0’ is not in phase with the gate input signal, and therefore the real measurement time is a random value ${\tilde{n}}_{g}/f_{x}$, with ${\tilde{n}}_{g}\in \left(n_{g}-1,\;n_{g}\right]\subset \mathbb{R}$.

On the other side, neglecting the stochastic contribution of electronic noise, jitter, clock instability and the phase displacement between the input signal and the reference clock, the pulse counter output $n_{p}$ is the truncation of the product between the measurement time and the reference clock frequency:

(13) $${\tilde{n}}_{p}=\frac{n_{g}}{f_{x}}f_{clk}\;.$$

${\tilde{n}}_{p}$ is a real number, defined in the range $\left[n_{p},\;n_{p}+1\right)$.

Putting together the counting uncertainty of the gate and the pulse counters, according to (12) we get that the input signal frequency $f_{x}$ is limited in the following interval:

(14) $$\frac{n_{g}-1}{n_{p}+1}f_{clk}<f_{x}\leq \frac{n_{g}}{n_{p}}f_{clk}\;,$$

therefore, the maximum count error corresponds to an error on the frequency measurement equal to:

(15) $$e_{max}\left({\hat{f}}_{x}\right)=\frac{n_{g}}{n_{p}}f_{clk}-\frac{n_{g}-1}{n_{p}+1}f_{clk}=\frac{n_{g}+n_{p}}{n_{p}\left(n_{p}+1\right)}f_{clk}\;.$$

Observing that in general $f_{clk}\gg f_{x}$, we have that $n_{p}\gg n_{g}$, therefore (11) can be approximated as:

(16) $$e_{max}\left({\hat{f}}_{x}\right)\approx \frac{n_{p}}{n_{p}^{2}}f_{clk}=\frac{f_{clk}}{n_{p}}=\frac{{\hat{f}}_{x}}{n_{g}}\approx \frac{f_{x}}{n_{g}}\;.$$

According to (16), the maximum measurement error depends only on the measurement time, regardless of the characteristics of the pulse counter. What is more, for short measurement times and input frequencies much greater than the required measurement accuracy, the resulting error can be high with respect to the desired accuracy.

**Figure 3:**
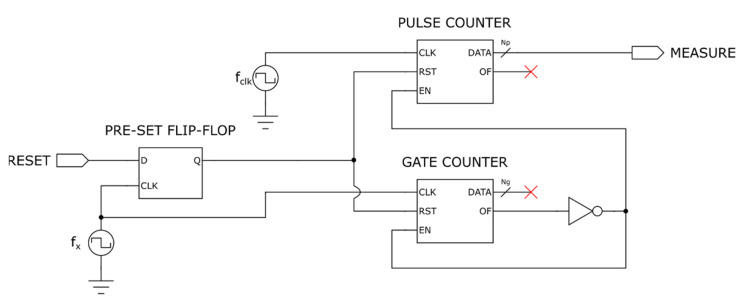
Structure of an equal precision reciprocal frequency counter.

To overcome these problems, we can employ a slightly different frequency counter architecture with respect to the one shown in Figure 2, called equal precision frequency meter [61], whose structure is shown in Figure 3.

In this architecture, the RESET signal is given as input to a D Flip-Flop synchronized with the gate signal, rather than being directly connected to the pulse and the gate counters; in this way, the counters enable instant is delayed with respect to the RESET switching instant to the first rising edge of the gate signal, granting the measurement time to be exactly equal to $n_{g}/f_{x}$ and reducing the maximum count error of the frequency counter to:

(17) $$e_{max}\left({\hat{f}}_{x}\right)=\frac{n_{g}}{n_{p}}f_{clk}-\frac{n_{g}}{n_{p}+1}f_{clk}=\frac{n_{g}}{n_{p}\left(n_{p}+1\right)}f_{clk}\approx \frac{n_{g}}{n_{p}^{2}}f_{clk}\;.$$

Putting together (8) and (13), we finally get:

(18) $$e_{max}\left({\hat{f}}_{x}\right)\approx \frac{1}{n_{g}}\frac{{\hat{f}}_{x}^{2}}{f_{clk}}\approx \frac{1}{n_{g}}\frac{f_{x}^{2}}{f_{clk}}\;.$$

Apparently, if we consider a limited measurement time application such as the QCM-D technique, in which the measurement time is upper bounded by the QCM transient response duration, the count error reported in (18) represents a performance limitation that cannot be overcome, as it is the result of a numerical truncation. In practice, however, it is possible to further reduce the error by averaging repeated measurements, exploiting the initial phase shift between the input signal and the clock signal.

**Figure 4:**
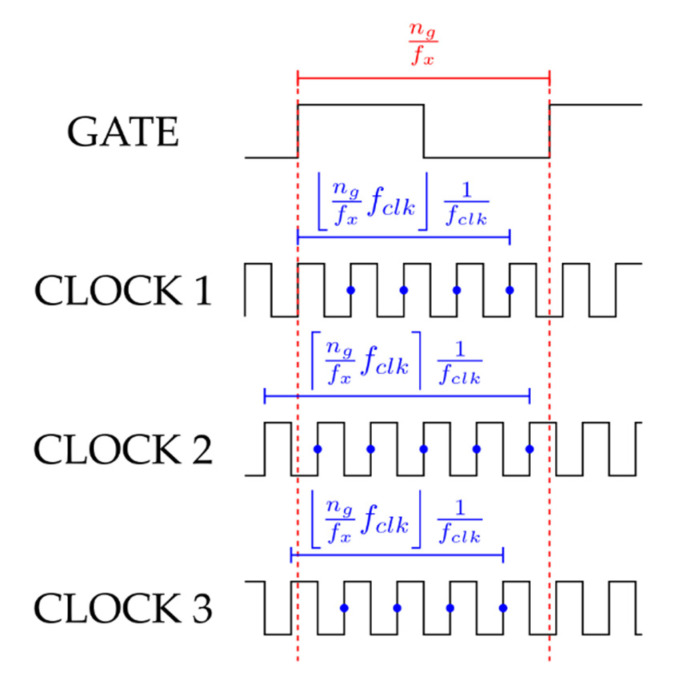
Graphical representation of the frequency meter output dependency on the phase difference between the input and the clock signals. CLOCK 1 is a clock in phase with the input signal, for which the frequency count is equal to the floor of the product ${\tilde{n}}_{p}$ of the measurement time $n_{g}/f_{x}$ and the clock frequency $f_{clk}$. CLOCK 2 is a clock delayed with respect to the input signal, with a delay smaller than the truncation error (${\tilde{n}}_{p}T_{clk}-⎣{\tilde{n}}_{p}⎦T_{clk})$, for which the frequency count is equal to the ceil of ${\tilde{n}}_{p}$. CLOCK 3 is a clock delayed with respect to the input signal, with a delay greater than the truncation error (${\tilde{n}}_{p}T_{clk}-⎣{\tilde{n}}_{p}⎦T_{clk})$, for which the frequency count reverts to the floor of ${\tilde{n}}_{p}$.

In (13) we stated that the frequency meter output is a count value $n_{p}$ corresponding to the floor of the product ${\tilde{n}}_{p}$ between the measurement time $n_{g}/f_{x}$ and the clock frequency $f_{clk}$; however, this statement is true only when the gate and the clock signals are initially in phase, i.e., if the measurement window starts with a rising edge of both the input and the clock signals; in this way, each clock count corresponds to a period equal to $T_{clk}=1/f_{clk}$. Conversely, if the two signals are not in phase, i.e., the first clock rising edge is delayed with respect to the measurement start instant, the first clock count occurs after a time shorter than $T_{clk}$, although the frequency meter interprets it as a whole clock period; as a consequence, if the clock delay is smaller than the fractional part of ${\tilde{n}}_{p}$ times $T_{clk}$ (the truncation error, i.e., ${\tilde{n}}_{p}T_{clk}-⎣{\tilde{n}}_{p}⎦T_{clk}$), the resulting frequency meter output count becomes the ceil of ${\tilde{n}}_{p}$; this effect is depicted by the three examples shown in Figure 4.

Since the equal precision reciprocal frequency meter is always synchronized with the input signal, without any synchronization with the clock signal, the clock delay can be considered a uniformly distributed random variable defined between 0 and $T_{clk}$; it can be easily proven that this random contribution to the frequency count allows, on average, to recover the input frequency removing the count error.

Let us consider again the product of the measurement time and the reference clock frequency ${\tilde{n}}_{p}$. As previously stated, this is a deterministic real number defined in the range $\left[n_{p},\;n_{p}+1\right)$, being $n_{p}$ the floor of ${\tilde{n}}_{p}$. Normalizing with respect to the clock period $T_{clk}$, the phase displacement between the input signal and the clock can be represented as a random variable $\Delta n_{p}$ uniformly distributed between 0 and 1. As a consequence, actually, the frequency meter output count is the result of the truncation of the random variable ${\tilde{n}}_{p}^{\prime }={\tilde{n}}_{p}+\Delta n_{p}$ uniformly distributed between ${\tilde{n}}_{p}$ and ${\tilde{n}}_{p}+1$, i.e., defined in the range $\left[n_{p},\;n_{p}+2\right)$.

This implies that the frequency meter output count is a discrete random variable $n_{p}^{\prime }$ defined on the set $\left\{n_{p},n_{p}+1\right\}$, with the probabilities of the following symbols:

(19) $$\begin{array}{c} P\left(n_{p}\right)=\int_{n_{p}}^{n_{p}+1}f\left({\tilde{n}}_{p}^{\prime }\right)d{\tilde{n}}_{p}^{\prime }=\int_{{\tilde{n}}_{p}}^{n_{p}+1}d{\tilde{n}}_{p}^{\prime }=n_{p}+1-{\tilde{n}}_{p}\\ P\left(n_{p}+1\right)=\int_{n_{p}+1}^{n_{p}+2}f\left({\tilde{n}}_{p}^{\prime }\right)d{\tilde{n}}_{p}^{\prime }=\int_{n_{p}+1}^{{\tilde{n}}_{p}+1}d{\tilde{n}}_{p}^{\prime }={\tilde{n}}_{p}-n_{p} \end{array}\;,$$

where $f\left({\tilde{n}}_{p}^{\prime }\right)$ is the probability density function of ${\tilde{n}}_{p}^{\prime }$, defined as:

(20) $$f\left({\tilde{n}}_{p}^{\prime }\right)=\left\{\begin{array}{cc} \;1 & \mathrm{if}\;{\tilde{n}}_{p}^{\prime }\in \left[{\tilde{n}}_{p},{\tilde{n}}_{p}+1\right)\\ \;0 & \mathrm{otherwise} \end{array}\right.\;.$$

It can be observed that the expected value of$\;n_{p}^{\prime }$ is equal to:

(21) $$\begin{array}{cc} E\left[n_{p}^{\prime }\right] & =n_{p}P\left(n_{p}\right)+\left(n_{p}+1\right)P\left(n_{p}+1\right)=\\ & =\;n_{p}\left(n_{p}+1-{\tilde{n}}_{p}\right)+\left(n_{p}+1\right)\left({\tilde{n}}_{p}-n_{p}\right)={\tilde{n}}_{p}\;, \end{array}$$

i.e., the average value of $n_{p}^{\prime }$ is equal to the exact product between measurement time and clock frequency.

Accordingly, averaging repeated measurements of a certain input frequency $f_{x}$ allows to reach error values lower than the maximum error provided in (18). What is more, the average is beneficial also with respect to other error contributions, such as jitter and electronic noise, being typically gaussian distributed with zero mean.

### 3.1. Equal Precision Reciprocal Frequency Counter Design Procedure

As shown in Figure 3, the equal precision reciprocal frequency counter is a very simple device, which requires, to be designed, only to choose the counters bit length.

Assume that we want to design a frequency counter to perform the frequency measurement of an unloaded QCM with transient oscillating frequency $f_{q}$; according to the sensing application, when the quartz is loaded by the adlayer or by the surrounding medium, this frequency is reduced, and we consider as the minimum possible frequency for the given application $f_{q}-\Delta f_{q}$; moreover, we assume the QCM-D decaying response minimum time duration equal to $T_{0}\approx 5\tau_{min}$, being $\tau_{min}$ the minimum decay time. Suppose that the device on which we are implementing the frequency counter works with a clock frequency equal to $f_{clk}$.

The gate counter bit length $N_{g}$ must be chosen big enough to grant that the counter reaches overflow in $T_{0}$ for any possible frequency of the quartz. In other words, we must size the counter with respect to $f_{q}-\Delta f_{q}$, choosing the minimum integer value of $N_{g}$ that allows setting the frequency counter measurement time greater or equal then $T_{0}$:

(22) $$N_{g}=\min\left\{N\in \mathbb{N}:\frac{2^{N}}{f_{q}-\Delta f_{q}}\geq T_{0}\right\}=\;⎡\log_{2}\left(\left(f_{q}-\Delta f_{q}\right)T_{0}\right)⎤\;.$$

In this way, we can configure the gate counter to count the maximum number of input signal periods $n_{g,max}$ observable in the measurement time:

(23) $$n_{g,max}=\max\left\{n\in \mathbb{N},n\leq 2^{N_{g}}:\frac{n}{f_{q}-\Delta f_{q}}\leq T_{0}\right\}=⎣\left(f_{q}-\Delta f_{q}\right)T_{0}⎦\;.$$

Along with $f_{clk}$, $N_{g}$ and $f_{q}-\Delta f_{q}$ define the maximum number of pulses $n_{p,max}$ we can count during the measurement time, i.e.,

(24) $$n_{p,max}=⎡\frac{f_{clk}}{f_{q}-\Delta f_{q}}\;2^{N_{g}}⎤\;.$$

The pulse counter bit length $N_{p}$ must be chosen high enough to grant that the counter is able to count at least up to $n_{p,max}$ without reaching overflow:

(25) $$\begin{array}{cc} N_{p} & =\min\left\{N\in \mathbb{N}:2^{N}>n_{p,max}\right\}=⎣\log_{2}\left(n_{p,max}\right)+1⎦\\ & =⎣N_{g}\log_{2}\left(\frac{f_{clk}}{f_{q}-\Delta f_{q}}\right)+1⎦\;. \end{array}$$

## 4. Application Case

To evaluate the performance of a QCM-D system based on a digital frequency meter, we consider an AT-cut quartz crystal with a nominal resonance frequency equal to 10 MHz mounted between two gold electrodes having a diameter of 6 mm. For such quartz, using an optimized electronic interface [16], the transient duration $T_{0}$ is about 1.6 ms in gas and 200 μs in water. Hence, considering in-liquid applications we consider $T_{0}\;$equal to 200 μs. Taking as a target application the real-time monitoring of biofilm growth through QCM, the implemented frequency meter should be capable of measuring frequency variations of the order of some Hz [38].

The frequency meter is implemented on a Xilinx Artix 7 xc7a45 FPGA, working at a frequency of 100 MHz, nominally generated by quartz with a stability of 50 ppm mounted on the FPGA board (Digilent Arty). Using a Mixed-Mode Clock Manager (MMCM) module, the FPGA frequency can be boosted up to 462.5 MHz.

The provided parameters can be used to properly size the frequency meter counters following the previously described procedure.

Setting the measurement time equal to the minimum signal duration, according to Equations (22) and (23) we get that the gate counter length must be:

(26) $$N_{g}=⎡\log_{2}\left(f_{q}T_{0}\right)⎤=⎡\log_{2}\left(10\;\mathrm{MHz}\cdot 200\;\mu s\right)⎤=11\;\mathrm{bit}\;,$$

with the gate count limited to:

(27) $$n_{g,max}=⎣f_{q}T_{0}⎦=⎣10\;\mathrm{MHz}\cdot 200\;\mu s⎦=2000\;.$$

Accordingly, applying Equations (24) and (25), we get that the maximum number of counted pulses is:

(28) $$n_{p,max}=\frac{f_{clk}}{f_{q}}\;2^{N_{g}}=\frac{462.5\;\mathrm{MHz}}{10\;\mathrm{MHz}}\;2^{11}=94720\;.$$

requiring a counter with a length equal to:

(29) $$N_{p}=⎣\log_{2}\left(n_{p,max}\right)+1⎦=17\;\mathrm{bit}\;.$$

Unfortunately, applying (18) to check the expected count error of a frequency meter of this kind:

(30) $$e_{max}\left({\hat{f}}_{x}\right)\approx \frac{1}{n_{g,max}}\frac{f_{q}^{2}}{f_{clk}}=\frac{1}{2000}\cdot \frac{{\left(10\;\mathrm{MHz}\right)}^{2}}{462.5\;\mathrm{MHz}}=108\;\mathrm{Hz}\;,$$

we notice that the frequency meter performance does not match the required measurement accuracy of some Hz. On the other hand, to keep the frequency meter count error lower than, for instance, 1 Hz, the input signal frequency should be lower, as derived from the following equation:

(31) $$e_{max}\left({\hat{f}}_{x}\right)\approx \frac{1}{⎣f_{q}T_{0}⎦}\frac{f_{q}^{2}}{f_{clk}}\geq \frac{1}{f_{q}T_{0}}\frac{f_{q}^{2}}{f_{clk}}\Rightarrow f_{q}\leq T_{0}f_{clk}e_{max}=92.5\;\mathrm{kHz}\;.$$

A condition of this kind can be achieved by performing proper preprocessing operations on the input signal before executing the frequency measurement. A possible approach to pass from the original QCM resonance frequency to a frequency value granting the desired measurement resolution is to down-shift the frequency of the QCM signal by mixing it with a signal with an appropriate frequency to reach the frequency band indicated in (31); this mixing operation can be performed in two ways:

- analog mixing;
- digital mixing, i.e., under-sampling.

Both techniques are well-known methods to perform the frequency difference between two periodic signals. From our application point of view, the main difference between the two techniques is the operation domain: the first technique is a purely analog technique, which requires analog processing of the QCM signal before it is provided as an input to the FPGA; conversely, under-sampling is a mixed-signal technique which can be partially performed in FPGA, 1-bit converting the QCM signal outside the FPGA and sampling the obtained square wave in the FPGA simply using a D Flip-Flop.

## 5. Measurement System Architecture

To test the proposed frequency meter, we realized a testbench that conditions the QCM transient response, preprocesses the QCM signal by either analog mixing or under-sampling, generating the mixing signal both with the FPGA or with an external waveform generator (Rigol DG4162), and finally performs the frequency measurement.

To the aim of obtaining a metrological characterization of the different frequency measurement techniques, in the realized test bench the QCM was emulated by means of an additional signal generator (Rigol DG4162).

This allows for testing the system with a source generating a ‘known’ oscillation frequency (characterized by high accuracy and stability). In fact, assessing the uncertainty of the proposed methods using a real QCM, by comparing the measured results with those obtained by theoretical predictions based on modeling, will introduce additional and larger sources of uncertainty (e.g., model accuracy).

**Figure 5:**
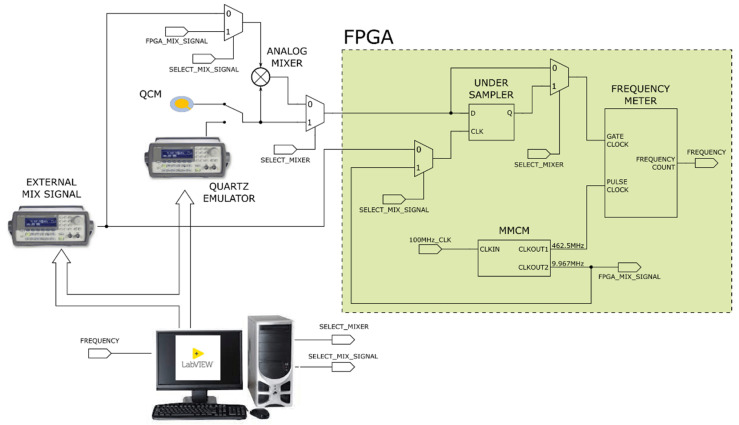
Functional block diagram of the frequency meter testbench.

Figure 5 shows the functional block diagram of the testbench.

A LabVIEW Virtual Instruments (VI) performs the testbench configuration selecting the mixing technique and the mixing signal.

Regardless of the preprocessing signal operation, the frequency meter performs the measurement on its input signal and provides the result to the VI for storing, postprocessing and visualization.

### 5.1. Experiments and Results

We employed the testbench to compare the performance of the frequency meter assuming to preprocess the quartz signal following four different approaches:

1. the QCM response signal is mixed using the analog mixer with another analog periodic signal (analog mixer + waveform generator signal) and then fed to the frequency meter;
2. the QCM response signal is mixed using the analog mixer with a periodic signal coming from the FPGA (analog mixer + MMCM signal) and then fed to the frequency meter;
3. the QCM response signal is under-sampled and quantized using a D Flip-Flop synchronized with an external signal generated by a waveform generator (under-sampler + waveform generator signal);
4. the QCM response signal is under-sampled and quantized using a D Flip-Flop synchronized with an internally generated FPGA signal (under-sampler + MMCM signal).

The tests were realized emulating different QCM frequencies in the range [9.998 MHz, 10 MHz] with steps of 1 Hz. The QCM signal was mixed with a 9.968 MHz periodic signal (generated by the FPGA or by the waveform generator), nominally providing mixed signals with frequencies ranging from 30 kHz to 32 kHz. The mixed signal was provided as input to the frequency meter. Finally, the measured frequency was compared with the ‘true’ (nominal) mixed signal frequency to evaluate the measurement error.

**Figure 6:**
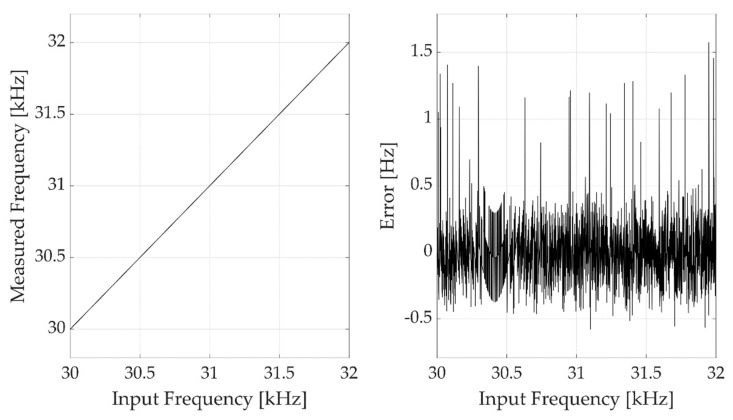
Measurement results observed directly providing to the FPGA digital frequency meter input square waves with fundamental frequencies in the range from 30 kHz to 32 kHz with a frequency step of 1 Hz, generated by a Rigol DG4162 waveform generator. The left plot shows the measured frequencies, the right plot shows the measurement error.

Considering the new input frequencies range and repeating the design procedure described in Section 3.1, the gate counter length was set at 3 bits, with a gate count $n_{g}$ equal to 6. With this configuration, the maximum expected count error was equal to 0.33 Hz. Figure 6 reports the results obtained by testing the frequency meter directly providing square waves with fundamental frequencies in the range of 30 kHz to 32 kHz generated by a waveform generator, with steps of 1 Hz and performing a single frequency measurement per frequency. As shown by the results in the figure, the measurement error is coherent with the expected one, despite being slightly higher because of the noise contributions due to the clock jitter and the FPGA on-board electronic noise. The results shown are corrected for the biasing related to the FPGA and signal generator clock accuracies.

**Figure 7:**
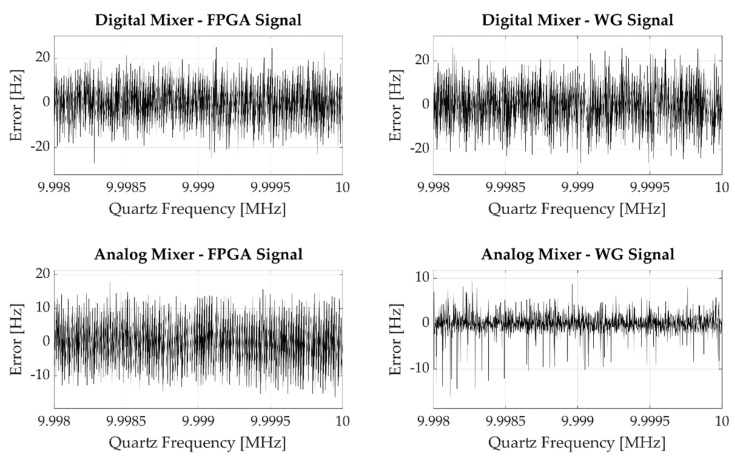
Error observed performing a single measurement for each generated frequency. The four plots show the results obtained by mixing the input signal using the digital or the analog mixer with the signal generated by the FPGA or by the waveform generator.

Having evaluated the effective measurement error of the frequency meter, we tested the four different architectures described above. The results obtained in the analyzed cases are shown in Figure 7, which pictures the experimental error observed performing a single measurement for each of the generated frequency; it can be easily observed that the resulting error is much higher than the one observed in Figure 6.

This increment of error is due to one of two possible causes, which are analyzed hereafter, depending on the used mixing technique.

**Figure 8:**
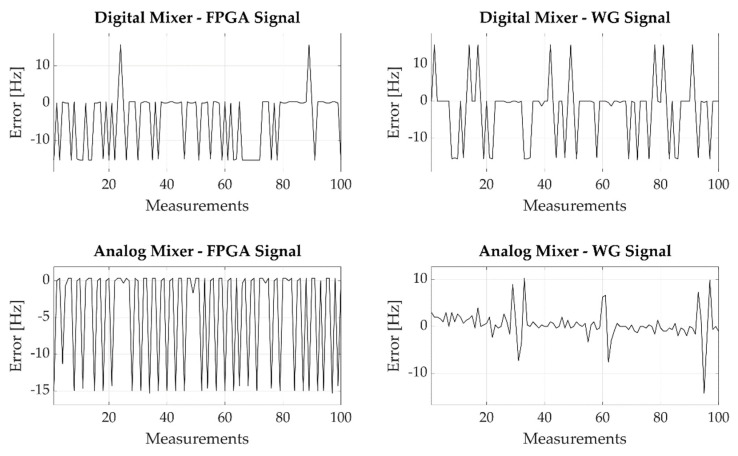
Experimental error observed performing 100 measurements setting the input frequency equal to 9.998 MHz. The four plots show the results obtained by mixing the input signal using the digital or the analog mixer along with the signal generated by the FPGA or by the waveform generator.

If we perform repeated measurements on a single input frequency, we get different error distributions according to the employed mixing process, as shown in Figure 8.

Focusing on the purely digital (Digital Mixer—FPGA Signal) measurement system and on the measurement system employing a purely analog front end (Analog Mixer—WG Signal) cases, we notice that the first solution produces quantized error levels, while the second one gives a more continuously distributed error.

In the analog case, the observed error depends on the fact that even if theoretically we use the mixer to generate a periodic signal whose frequency $f_{x}$ is the difference between the quartz frequency $f_{q}$ and the mixing frequency $f_{mix}$, in practice $f_{x}$ is affected by a certain jitter $\Delta f_{x}$ coming from the combination of the input signals jitters and the front-end electronic noise, i.e., $f_{x}=f_{x0}+\Delta f_{x}$, where $f_{x0}$ is the nominal signal frequency.

Consequently, the frequency meter count $n_{p}$ is the truncation of a uniformly distributed random variable, which can be described as:

(32) $$\begin{array}{c} {\tilde{n}}_{p}=\frac{f_{clk}}{f_{x0}+\Delta f_{x}}n_{g}=\frac{f_{clk}}{f_{x0}}n_{g}-\frac{\Delta f_{x}}{f_{x0}(f_{x0}+\Delta f_{x})}f_{clk}n_{g}\approx \frac{f_{clk}}{f_{x0}}n_{g}-\frac{\Delta f_{x}}{f_{x0}^{2}}f_{clk}n_{g}=\\ \;={\tilde{n}}_{p0}+\Delta {\tilde{n}}_{p}\;, \end{array}$$

where ${\tilde{n}}_{p0}$ is the nominal count and $\Delta {\tilde{n}}_{p}$ is the count variability.

Conversely, in the digital case we observe a quantized and higher error because of the sampling operation performed by the digital mixer. Since the digital mixer is realized through a Flip-Flop which performs the quartz signal under-sampling at the frequency $f_{mix}$, its output signal period $T_{x}\prime$ will be the truncated value of $T_{x}=1/f_{x}$ with respect to $T_{mix}=1/f_{mix}$, i.e.,:

(33) $$T_{x}^{\prime }=⎣\frac{T_{x}}{T_{mix}}⎦T_{mix}=kT_{mix}\;,$$

where k is a natural number; this means the digital mixer output signal period can be up to $T_{mix}$ shorter than the expected period, introducing a maximum count error equal to $⎣f_{clk}T_{mix}⎦$ counts. The combination of this count error with jitter, electronics noise, and phase displacement between the two signals ensures also in this case a statistical behavior, therefore the measurement falls onto quantized values with different probabilities functions whose mean value is related to the true frequency value, due to a behavior such as the one discussed in detail in Section 3.

Referring again to Figure 8, a final remark must be done for the results obtained by mixing the quartz signal with the FPGA-generated signal using the analog mixer. In this case, the resulting error is not distributed as uniformly as in the purely analog case because of the shape of the mixed signal. Since the signal coming from the FPGA is a square wave, unlike the signal generated by the waveform generator, which is a sine wave, the mixer output signal frequency error is still continuously distributed, but the mixing signal multiple integer frequencies are more probable with respect to the fractional ones.

In any case, regardless of the phenomena producing the mixer output signal frequency variability, the frequency meter performance can be improved simply by performing the average of repeated measurements.

**Figure 9:**
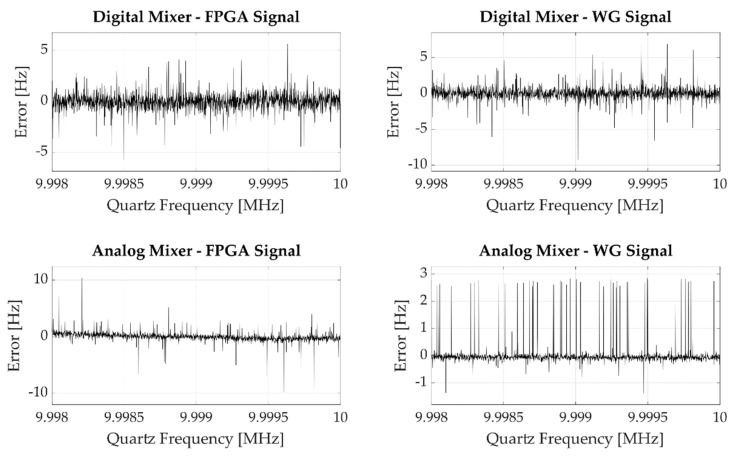
Experimental error observed while performing 100 measurements for each generated frequency. The four plots show the results obtained mixing the input signal using the digital or the analog mixer along with the signal generated by the FPGA or by the waveform generator.

Figure 9 shows the experimental error obtained by averaging the output of 100 repeated measurements for each input frequency. The number of averaged measurements is considered acceptable in the context of a QCM-D measurement technique, assuming to interrogate the quartz with a frequency of 100 Hz, i.e., a transient excited with a pulse repetition frequency (PRF) of 100 Hz, and to provide an estimation of the resonant frequency each second; this measurement time is reasonable for QCM-based monitoring in most applications where the system under test transients are usually in the tens of seconds or in the minutes ranges (e.g., bacterial growth, gas adsorption, film growth).

The average operation reduces the measurement error both in terms of maximum error and of standard deviation, as also reported in Table 1.

According to the obtained results, performing the average of 100 repeated measurements, the proposed architecture is characterized by a measurement error with zero mean (assuming calibrating the system to remove the biasing related to the FPGA and signal generator clock accuracies) and standard deviations, which are reduced, as expected, approximately by a factor $\sqrt{100}$, reaching values lower than 1 Hz. Comparing the four proposed architectures, the best performance is achieved by the purely analog and purely digital preprocessing solutions. Although the analog solution is the one with the best performance at all, the slightly higher error of the digital solution is compensated by a lower implementation complexity, as it is integrated into the digital architecture within the FPGA.

### 5.2. QCM Tests

Having characterized the frequency meter accuracy by using a waveform generator used as a QCM emulator, we applied the proposed frequency meter in a realistic application, i.e., the dynamic measurements in-liquid with a QCM whose characteristics corresponds to the application case proposed in Section 4, i.e., an AT-cut quartz crystal with nominal resonance frequency equal to 10 MHz mounted between two gold electrodes having a diameter of 6 mm, with expected in-liquid QCM-D transient duration of about 200 μs.

**Figure 10:**
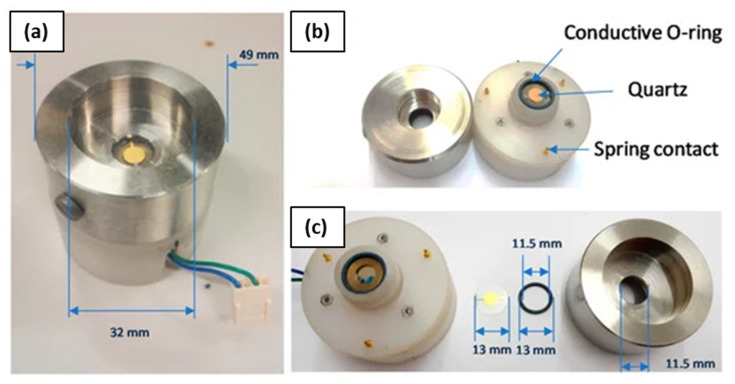
Measurement chamber used for the QCM tests. (**a**) shows the assembled chamber, (**b**,**c**) show the building blocks.

The QCM was placed in an ad-hoc-built measurement chamber [16]. The chamber, shown in Figure 10, is composed of two blocks, in the middle of which the QCM is sandwiched, working as a wall between them; the bottom block hosts a holder and a connector for the quartz, while the upper block presents a central hole which allows depositing liquid on the quartz when the chamber is closed. The bottom block is made of Teflon, while the top is realized in stainless steel which has been used due to the low interaction with chemical compounds. The contact with the quartz metallization is guaranteed by two conductive O-rings. An embedded copper ring in the Teflon structure is used to carry out the signal from the bottom metallization via the conductive O-ring. Concerning the top stainless steel structure, it is in electrical contact with the quartz top metallization via the conductive O-ring and it is then grounded at the front-end electronics input. The electrical contact between the top and the bottom is performed by spring contacts. The design of the chamber grants that the deposited liquid remains only on the upper surface of the quartz; in this way, it is possible to perform in-liquid measurements.

Moreover, the use of Teflon as the bottom block guarantees a reduction of parallel parasitic capacitances to the quartz crystal, which can affect the measurement results.

The QCM was excited by means of a waveform generator and its QCM-D response was processed by means of dedicated front-end electronics to be amplified and compared, to convert it into a square wave with a voltage range compatible with the FPGA. The obtained signal was than given as input to the architecture presented in Section 5.

**Figure 11:**
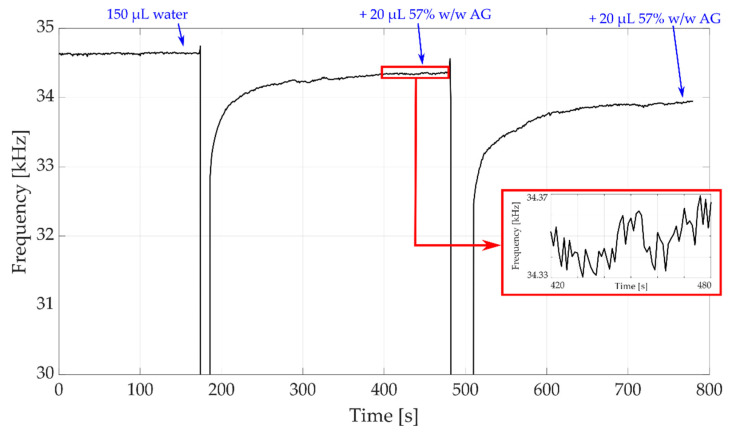
QCM monitoring starting from pure water (150 μL) and adding subsequent doses of 10 μL of a solution with 60% anhydrous glucose (AG) with a micropipette.

The presented system was used to monitor the QCM series resonating frequency in liquid. We started from pure water (150 μL) and then we added, with a micropipette, subsequent doses of 20 μL of a solution of water with 57% weight/weight (*w*/*w*) concentration of anhydrous glucose, obtaining solutions with anhydrous glucose *w*/*w* concentrations of 0%, 9.11% and 15.71%, corresponding to heavily loaded damped responses of the QCM, as they correspond to values of the resistance $R_{m}$ up to 210 Ω [2,48]; the observed frequency variations are reported in Figure 11.

Figure 11 shows the results obtained monitoring the QCM frequency for 13 min employing the proposed frequency meter with the analog mixer and the mixing signal generated by a waveform generator, without performing averages. The QCM-D pulse repetition frequency was set at 1 Hz, providing one measurement per second. The single measurement time was set equal to 200 μs.

The application of 10 μL of the solution of water with 60% anhydrous glucose causes a frequency shift of the QCM of around 200 Hz: in fact, at the end of the transient induced by the application of the solution with the micropipette, whose duration is of the order of few minutes, the mixed frequency value passes from an initial value of 34.65 kHz (pure water) to 34.45 kHz after the first solution dose application, and to 34.25 kHz after the second solution dose application; these results are in perfect accordance with what expected from the theory [38]; it can be observed, also looking at the figure, that the employed frequency meter achieves a frequency resolution of few Hertz, as expected from the previously performed characterization. Consequently, the performed measurements show that the proposed architecture can be employed to measure frequency shifts in QCM-D applications, since it can reach sufficient resolutions for the target applications, related to liquid density/viscosity assessment; this resolution can be further enhanced by averaging, if needed by the application, as in the case of biosensors.

Always referring to Figure 11, a final comment must be provided regarding the frequency meter behavior during the transient induced by solution application on the QCM; it can be observed that, at the beginning of the transient, the output frequency drops to 0; this effect is caused by the perturbation generated by the application of the solution: the solution is dropped manually with a micropipette; since the added dose has a different density and temperature with respect to the one on the QCM, when it falls on the QCM, a fluid dynamical transient is generated, during which the QCM-D response signal is so short in time to not be detectable by the frequency meter, as set for this simple test with fixed time gate duration, therefore producing the 0 Hz output.

## 6. Conclusions

We have proposed an FPGA-based measurement system for the monitoring of QCM oscillating frequency in QCM-D applications, tailored to in-liquid applications, in which the large mechanical loading reduces the duration of the quartz transient responses duration down to a few hundreds of μs, corresponding to a Q factor of about some thousands.

We have derived the proposed measurement circuit from the study of the theoretical count error introduced by an architecture of this kind, identifying the most suitable signal preprocessing technique to achieve the desired measurement resolution (in the order of a few ppb). We proposed different low-cost circuits implementing four different preprocessing techniques, based on the mixing of the quartz signal to increase the frequency meter resolution, exploiting both analog and digital hardware.

The four circuits’ performance was tested experimentally with the use of a test bench capable of emulating the QCM-D response signal with selected frequencies. The experimental results showed that the effect of noise and implementation non-idealities can significantly decrease the system performance, especially due to the implementation of down-shifting in frequency by means of under-sampling, as required by the all-digital solution; this effect is related to the system main clock frequency and depends on the ratio between this frequency and the QCM resonance one. The additional errors behave as white noise signals. Therefore, it was shown that averaging subsequent measurements mitigate the issue, providing satisfactory results. In particular, the experimental results concerned the operations with 10 MHz QCMs loaded by water solutions and characterized by a transient response lasting at least 200 μs. The proposed measurement system settings were adjusted to achieve a nominal frequency resolution of 0.3 Hz whereas the actual resolution was experimentally estimated to be 24 Hz (3 sigmas of the noise floor) for the all-digital solution and 6 Hz for the circuit embedding an analog mixer and a DDS based generation of the mixing signal. Passing through average operations of N subsequent measurement (processing N subsequent transient responses) encompassing a window where the process under measurement can be considered stationary the additional noise can be reduced by about a factor $\sqrt{N}$. In the explored experimental cases the stationary window was assumed to be 1 s, with a PRF of 100 Hz that allows setting N to be equal to 100; under this setup, the obtained resolution reached by the low-cost full-digital solution is about 2.4 Hz.

In summary, this work showed that the main sources of uncertainty are white zero mean processes, therefore increasing the measurement time and averaging, when possible, allows for lower uncertainty and improved resolution: longer times come both from increasing the number of pulse repetitions used for averaging and from using a longer gate. Nevertheless, the length of the gate is limited by the duration of the transient, whereas the number of repetitions is limited by the stationarity of the observed phenomenon.

Finally, we tested the proposed architecture by performing dynamical measurements on a QCM, monitoring its frequency variation in liquid while changing the applied load. The resulting measurement accuracy appears to be fully compatible with the QCM-D application, being the observed uncertainty more than one order of magnitude smaller than the frequency variations, confirming therefore the validity of the proposal.

## Figures and Tables

**Table 1:** Quartz mixing signal topologies comparison in terms of maximum error and error standard deviation performing a single measurement on the frequency meter input signal and averaging 100 repeated measurements performed on each frequency.

Topology	Single Measurement Error	Averaged Measurements Error
Digital Mixer	$e_{max}=27\;\mathrm{Hz}$	$e_{max}=6\;\mathrm{Hz}$
FPGA Signal	$\sigma \left(e\right)=8.3\;\mathrm{Hz}$	$\sigma \left(e\right)=0.8\;\mathrm{Hz}$
Digital Mixer	$e_{max}=27\;\mathrm{Hz}$	$e_{max}=10\;\mathrm{Hz}$
WG Signal	$\sigma \left(e\right)=9.1\;\mathrm{Hz}$	$\sigma \left(e\right)=0.9\;\mathrm{Hz}$
Analog Mixer	$e_{max}=18\;\mathrm{Hz}$	$e_{max}=11\;\mathrm{Hz}$
FPGA Signal	$\sigma \left(e\right)=6.4\;\mathrm{Hz}$	$\sigma \left(e\right)=0.8\;\mathrm{Hz}$
Analog Mixer	$e_{max}=17\;\mathrm{Hz}$	$e_{max}=3\;\mathrm{Hz}$
WG Signal	$\sigma \left(e\right)=2.2\;\mathrm{Hz}$	$\sigma \left(e\right)=0.4\;\mathrm{Hz}$

## Data Availability

Not applicable.
